# Regulation of PPAR*γ* Coactivator-1*α* Function and Expression in Muscle: Effect of Exercise

**DOI:** 10.1155/2010/937123

**Published:** 2010-08-19

**Authors:** Giulia Uguccioni, Donna D'souza, David A. Hood

**Affiliations:** ^1^School of Kinesiology and Health Science, York University, Toronto, ON, Canada M3J 1P3; ^2^Muscle Health Research Centre, York University, Toronto, ON, Canada M3J 1P3; ^3^Department of Biology, York University, Toronto, ON, Canada M3J 1P3

## Abstract

PPAR*γ* coactivator-1*α* (PGC-1*α*) is considered to be a major regulator of mitochondrial biogenesis. Though first discovered in brown adipose tissue, this coactivator has emerged as a coordinator of mitochondrial biogenesis in skeletal muscle via enhanced transcription of many nuclear genes encoding mitochondrial proteins. Stimuli such as exercise provoke the activation of signalling cascades that lead to the induction of PGC-1*α*. Posttranslational modifications also regulate the function of PGC-1*α*, with a multitude of upstream molecules targeting the protein to modify its activity and/or expression. Previous research has established a positive correlation between resistance to fatigue and skeletal muscle mitochondrial content. Recently, studies have begun to elucidate the specific role of PGC-1*α* in exercise-related skeletal muscle adaptations, with several studies identifying it as a dominant regulator of organelle synthesis. This paper will highlight the function, regulation, and expression of PGC-1*α*, as well as the role of the coactivator during exercise adaptations.

## 1. Introduction

The PPAR*γ* coactivator-1 (PGC-1) family of coactivators is a well studied group of coregulatory proteins. These proteins are classified as second-class coactivators and consist of PPAR*γ* coactivator-1*α* (PGC-1*α*), -1*β* (PGC-1*β*), and PGC-1-related coactivator (PRC). All three PGC-1 members share a basic protein structure consisting of an activation domain with the LXXLL sequence in the N-terminal, a central region associated with repression, and a RNA-binding motif (RMM) and serine-arginine-rich (RS) region in the C-terminal region [1]. Collectively, PGC-1 coactivators have emerged as major regulators of multiple cellular processes, including adaptive thermogenesis, glucose metabolism, and muscle fiber type specialization, and they are considered to be predominantly involved in the regulation of mitochondrial content and function [2–6]. While all three PGC-1 family members are implicated in the regulation of mitochondrial biogenesis, PGC-1*α* is the most well-characterized of the group. PGC-1*β* was first discovered based on its sequence similarity to PGC-1*α* [7, 8], and much research on PGC-1*β* has been focused on its function specifically in relation to PGC-1*α*, in particular it's involvement in the regulation of mitochondrial content and oxygen respiration [9]. Conversely, the role of PRC remains to be well-defined. Research thus far has also implicated PRC in the regulation of mitochondrial respiratory function, along with a role in cell cycle progression, whereby PRC is upregulated in proliferating cells, and responsive to serum growth factors [10–12]. In contrast, a multitude of studies have revealed a dominant role of PGC-1*α* in the regulation of mitochondrial content, and based on the plethora of data to support this, it is not surprising that the coactivator is currently considered one of the most important regulators of mitochondrial biogenesis [4].

## 2. PGC-1*α* Function

PGC-1*α* is the founding member of the PGC-1 family of coactivators that was originally identified based on its functional interaction with the transcription factor PPAR*γ* in brown adipose tissue [4]. The gene for PGC-1*α* is located on chromosome four in humans (chromosome five in mice) and encodes a protein consisting of 798 amino acids (797 amino acids in mice) with a predicated molecular weight of 92 kDa [13]. 

PGC-1*α* does not contain intrinsic enzymatic activities, rather several proteins containing histone acetyltransferase (HAT) activity bind to the activation domain on the N-terminal of PGC-1*α* when the coactivator is bound to a transcription factor. The binding of these proteins, such as p300, SRC-1, and CREB-binding protein (CBP) to PGC-1*α*, remodels histones on chromatin and allows for greater access of the transcriptional machinery to DNA for gene transcription [14]. Neighbouring the N-terminus is a 200 amino acid negative regulatory region that represses the function of the activation domain in PGC-1*α* [14]. At the C-terminus, proteins that bind, such as the TRAP/DRIP mediator complex, can aid in the displacement of repressor proteins such as histone deacetylases (HDACs) which function by deacetylating histones, thereby keeping DNA in a coiled state. The displacement of HDACs via the interaction of PGC-1*α* and the mediator complex allows for the relaxation of chromatin, greater access of transcription factors to DNA, and enhanced gene transcription [15]. The RNA-binding motif (RMM) and serine-arginine rich region (RS) domains in the C-terminus are characteristic of proteins with mRNA splicing and export activity [16]. It has been found that PGC-1*α* associates with RNA polymerase II during elongation and is also involved in the splicing and export of several mRNA products [16]. Thus, collectively PGC-1*α* acts as a platform to integrate many proteins and processes involved in the production of mature mRNA transcripts [4, 17]. 

Early studies examining the function of PGC-1*α* in brown adipose tissue suggested a role for PGC-1*α* in the regulation of mitochondrial content, termed mitochondrial biogenesis. For example, analysis of the expression pattern of PGC-1*α* across multiple tissues revealed strong correlations between oxidative capacity and protein levels of the coactivator [5, 16, 18, 19]. Protein and mRNA levels of PGC-1*α* are greatest in tissues with a high metabolic activity, such as brown fat, heart, and skeletal muscle [4, 19]. Since its discovery, there has been an abundance of research which has pointed out the importance of PGC-1*α* in regulating mitochondrial content [4, 5, 19, 20]. As noted below, PGC-1*α* is specifically involved in the maintenance of mitochondrial function, respiration, and content in skeletal muscle [4, 5, 20, 21]. 

**Figure 1:**
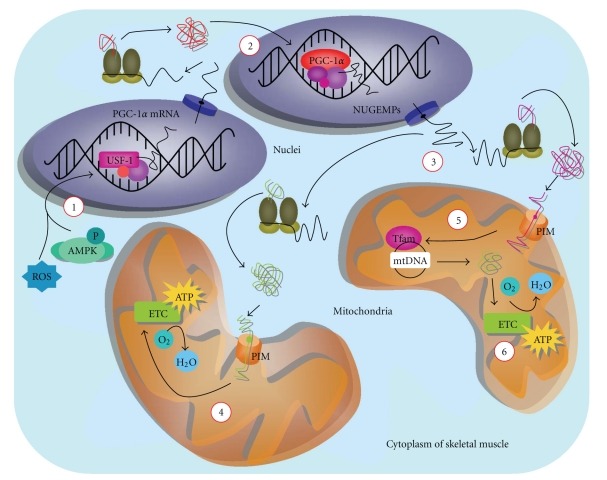
PGC-1*α* induces expression of nuclear and mitochondrial genes required for organelle synthesis. Expression of PPAR*γ* coactivator-1*α* is regulated by a number of transcription factors (e.g., USF-1, CREB) which associate with its promoter region to augment transcription. External stimuli, such as exercise, provoke the formation of reactive oxygen species (ROS) and activate AMPK. These signalling molecules induce PGC-1*α* transcription (1), and increase coactivator mRNA expression. Upon formation, the nascent transcript is transported to the cytoplasm. Once translated into protein, PGC-1*α* is sequestered to the nucleus (2), interacting with transcription factors to promote the transcription of nuclear genes encoding mitochondrial proteins (NUGEMPs). Specifically, PGC-1*α* is known to associate with nuclear respiratory factors 1 and 2 (NRF-1, NRF-2), guiding the transcription of mitochondrially-destined proteins. NUGEMP mRNA is exported to the cytoplasm and is subject to translation (3). The translated precursor proteins are imported into the mitochondria (4) via the protein import machinery (PIM). Many of these form multisubunit complexes that become part of the electron transport chain (ETC). One particular nuclear-derived protein is mitochondrial transcription factor A (Tfam). Once imported into the mitochondria, it associates with mitochondrial DNA (mtDNA) (5), directing the production of mtDNA-encoded proteins. These products are then integrated into the ETC (6). Thus, PGC-1*α* is an important protein which initiates a cascade of events leading to mitochondrial biogenesis.

 The process of mitochondrial biogenesis is complex because the expansion of the mitochondrial network requires the cooperation of both the nuclear and mitochondrial genomes. Mitochondria contain double-stranded circular DNA that encodes less than 1% of the proteins necessary for the electron transport chain (ETC) and organelle function [22, 23]. Thus, mitochondrial biogenesis is highly dependent on the expression of proteins encoded by nuclear DNA to ensure the proper assembly and expansion of the mitochondrial reticulum [24]. This effect is mediated in part by the ability of PGC-1*α* to coactivate many transcription factors that bind to the promoter region of nuclear genes encoding mitochondrial proteins (NUGEMPs; Figure 1). In particular, PGC-1*α* interacts with nuclear respiratory factors (NRF)-1 and -2 to transactivate genes involved in the electron transport chain (ETC), the protein import machinery, as well as transcription factors of mitochondrial DNA (mtDNA), such as mitochondrial transcription factor A (Tfam) [25, 26]. Recently, the estrogen-related receptor, ERR*α*, has also emerged as an important regulator of oxidative metabolism. ERR*α* binding sites are present on the promoters of several genes of the ETC, such as cytochrome c and *β*-ATP synthase [27]. Furthermore, ERR*α* activity is regulated by PGC-1*α*, because its ablation compromises the ability of PGC-1*α* to induce mitochondrial biogenesis [9]. It is also well established that PGC-1*α* coactivates the PPAR family of nuclear receptors, such as PPAR*δ*, which induces the expression of mitochondrial proteins (e.g., cytochrome c oxidase subunit II (COXII) and COXIV) that are important for biogenesis [28, 29]. Thus, the ability of PGC-1*α* to orchestrate the transcriptional activity of these, and several other nuclear transcription factors, allows the coactivator to coordinate the large number of genes essential for mitochondrial biogenesis.

## 3. Regulation of Expression

Alterations in the mitochondrial content of tissues such as skeletal muscle have a large impact on cellular metabolism and whole body health. Since PGC-1*α* is a vital regulator of mitochondrial content, it is therefore physiologically relevant to understand, in detail, the regulation of PGC-1*α* expression. PGC-1*α* is induced by multiple factors, such as environmental, hormonal, and nutritional stimuli across a variety of tissues [19, 30, 31]. In skeletal muscle, PGC-1*α* expression tends to coincide with signalling pathways typically activated with mitochondrial biogenesis, such as those induced by endurance exercise. These include the activation of p38 MAP kinase, AMPK, Ca^2+^-mediated pathways involving CaMK, as well as ROS-induced signaling. All have been mediated in the regulation of PGC-1*α* promoter activity. For example, activation of cAMP responsive element binding protein (CREB) and activating transcription factor 2 (ATF2) via either Ca^2+^/CaMKIV or p38 mitogen-activated protein kinase (MAPK), respectively, stimulate binding of CREB to the cAMP responsive element (CRE) in the promoter of PGC-1*α* [32, 33]. Indeed, overexpression of Ca^2+^-activated CaMK increases PGC-1*α* promoter activity [2], and treatment of muscle cells with caffeine to induce an increase in intracellular cytosolic Ca^2+^ results in an increase in PGC-1*α* expression which is dependent on CaMK activity [34]. Interestingly, the induction of PGC-1*α* in response to raised Ca^2+^ is most likely a result of the downstream activation of p38 by CaMK, because inhibiting the phosphorylation of p38 prevents increases in PGC-1*α* even in the presence of active CaMK. This effect is likely mediated by p38*γ*, not p38*α* or *β* [35]. These data suggest that physiological conditions which evoke elevations in cytosolic calcium, such as contractile activity, could augment PGC-1*α* expression via transcriptional activation of the PGC-1*α* promoter. 

Signals which activate AMPK also contribute to an increase in PGC-1*α* expression [19]. AICAR treatment, a known activator of AMPK, increased the mRNA levels of PGC-1*α* in myoblasts after only 24 hours of treatment. This was the result of an AMPK-mediated increase in the binding of upstream stimulatory factor-1 (USF-1) to a GATA/Ebox consensus sequence in the PGC-1*α* promoter [36]. H_2_O_2_ also provoked an increase in PGC-1*α* transcription, in part by H_2_O_2_-induced binding of USF-1 to the PGC-1*α* promoter, along with the activation of AMPK elicited by exogenous H_2_O_2_ levels [37]. Although these are the major pathways that appear to regulate the transcriptional activity of PGC-1*α*, the coactivator is also involved in an autoregulatory loop controlling its own level of activity. Some transcription factors that PGC-1*α* coactivates, such as myocyte enhancer factor (MEF)-2 and myogenic determining factor (MyoD), also bind to, and regulate, the PGC-1*α* promoter [38, 39]. Thus, there are multiple, likely overlapping and redundant, signaling pathways which mediate the transcription of this important metabolic coactivator.

## 4. Posttranslational Modifications of PGC-1*α*

Regulation of PGC-1*α* at the protein level via protein stability, subcellular localization or activation can also account for PGC-1*α*-mediated actions on mitochondrial biogenesis. The phosphorylation of PGC-1*α* by p38 MAPK was the first posttranslational modification that was identified which alters protein stability. In skeletal muscle, p38 MAPK phosphorylates of multiple serine/threonine residues (Thr262, Ser265 and Thr298). This results in an increase in PGC-1*α* protein levels as a consequence of increased protein stability from a half-life of 2.3 to 6.3 hours [40]. In contrast, phosphorylation by Akt on Ser570 results in an unstable protein with impaired activity [41]. p38 MAPK also has an additional effect that promotes the release of PGC-1*α* from inhibitory factors [42, 43]. Under steady state conditions, p160 myb binding protein (p160^MBP^) binds to, and represses, the transcriptional activity of PGC-1*α*. The p38-mediated phosphorylation of PGC-1*α* disrupts the binding of p160^MBP^ to the coactivator, allowing for its activation [42]. 

In addition to p38, additional kinases also phosphorylate and modulate the activity of PGC-1*α*. AMPK directly phosphorylates PGC-1*α* (Thr177 and Ser538), which is essential for the AICAR-dependent induction of the PGC-1*α* promoter [44]. This mechanism may potentially be involved in the mitochondrial adaptations, such as increases in cytochrome c and cellular respiration that are provoked by AICAR [45]. Thus, the activation of AMPK and its phosphorylation of downstream targets, such as PGC-1*α*, is likely important in the regulation of nuclear genes encoding mitochondrial proteins in response to exercise.

Additional posttranslational modifications of PGC-1*α* include acetylation and methylation of the protein. The NAD^+^-dependent histone deacetylase, silent information regulator 1 (SIRT1), interacts with PGC-1*α* and deacetylates the coactivator on various lysine residues between amino acids 200 and 400 [46, 47]. The deacetylation via SIRT1 keeps PGC-1*α* in an active state. In skeletal muscle, the activation of SIRT1 stimulates the upregulation of mitochondrial gene expression in a manner that is at least partly due to the deacetylation of PGC-1*α* [48]. Conversely, evidence from neural cells indicates that the deacetylation of PGC-1*α* by SIRT1 decreases activity of the coactivator [46]. This suggests that the effects of SIRT1 on the activity of PGC-1*α* occur in a tissue-specific manner. In contrast to deacetylation, the acetylation of PGC-1*α* by general control of amino acid synthesis 5 (GCN5) acetyltransferase results in a transcriptionally inactive protein [45]. This repression of PGC-1*α* is attributed to the acetylation-induced translocation of the coactivator to nuclear foci where it is inhibited by the transcriptional corepressor, nuclear receptor interacting protein 140 (RIP140). 

In addition to acetylation, lysine residues on PGC-1*α* can also be modified by small ubiquitin-like modifier (SUMO) proteins. The sumoylation of proteins has many functions that can affect protein subcellular localization, stability, activity, and capacity to interact with other proteins and activity [49]. PGC-1*α* can be sumoylated on lysine residue 183 on its activation domain [50]. The addition of SUMO proteins attenuates the transcriptional activity of PGC-1*α* by enhancing the sensitivity of PGC-1*α* to the repressive actions of RIP140, independent of any alterations in the localization or stability of the coactivator [50]. Further identification of the multitude of signals and modifications which regulate the activity of PGC-1*α* will continue to provide a better understanding of the function of this metabolic regulator.

## 5. The Effects of PGC-1*α* Overexpression and Knockout on Exercise Performance

The majority of research regarding PGC-1*α* has primarily involved the overexpression and/or ablation of the protein using animal and cell culture models. The ectopic expression of PGC-1*α* in cultured muscle cells induces large increases in mitochondrial content and augments both basal and active cellular respiration rates [5]. In fact, the overexpression of PGC-1*α* has repeatedly been shown to increase levels of NUGEMPs essential for mitochondrial biogenesis [2, 51]. For example, elevated levels of PGC-1*α* in C2C12 cells enhances nuclear- (e.g., cytochrome c, COX subunit IV) and mitochondrially- (e.g., COX subunit II) encoded components of the respiratory chain and amplifies mtDNA copy number [5]. These results have also been replicated *in vivo*. Both whole body and muscle-specific overexpressing PGC-1*α* animals display increases in markers of mitochondrial content along with overall increases in oxidative capacity. PGC-1*α* transgenic animals also exhibit physiological improvements in whole body oxygen uptake and decreased fatigue in response to an exercise stimulus [2, 51]. Altering the expression of PGC-1*α* *in vivo* has also revealed an important role of PGC-1*α* in muscle refuelling. Gain-of-function experiments resulted in increased muscle glucose uptake and decreased rates of glycolysis, along with concomitant increases in muscle glycogen stores and the prevention of glycogen depletion in response to exercise [52]. 

Conversely, PGC-1*α*-deficient animals also reveal the essential nature of PGC-1*α* for the normal expression of mitochondrial genes important for organelle biogenesis [53, 54]. The mRNA levels of both nuclear and mitochondrial genes are reduced in several tissues, including skeletal muscle [54]. Lack of PGC-1*α* also led to a reduced mitochondrial content, as revealed by COX activity in striated muscle, along with deficits in mitochondrial function. The ablation of PGC-1*α* significantly impaired mitochondrial respiration rates, indicating a diminished ability to produce ATP relative to wild-type (WT) control animals [20]. These alterations in mitochondrial content and function relate to the reduced endurance capacity observed in PGC-1*α* KO animals [55]. Collectively, the data indicate that the manipulation of PGC-1*α* has revealed a very dynamic role for the coactivator that appears to be centered on its involvement in the regulation of mitochondrial biogenesis.

## 6. Response to Exercise

It has long been known that exercise elicits changes in the expression of NUGEMPs and enhances the process of mitochondrial biogenesis in skeletal muscle. In fact, the discovery of PGC-1*α* gave rise to the possibility that a main regulatory protein could control the mitochondrial adaptations observed with endurance exercise, or in response to chronic contractile activity (CCA). Thus, PGC-1*α* has been readily implicated in mediating exercise-induced mitochondrial biogenesis. The use of chronic muscle stimulation and acute exercise paradigms have demonstrated increases in PGC-1*α* mRNA and protein levels [19, 31, 56]. As expected, these exercise models also resulted in the concurrent upregulation of genes encoded by both the nuclear and mitochondrial genomes involved in mitochondrial biogenesis [57, 58]. The coordinated changes in the expression of PGC-1*α* and NUGEMPs with exercise have founded the basis for a role of PGC-1*α* in exercise-induced mitochondrial adaptations in skeletal muscle.

The particular mechanisms underlying the increases observed in PGC-1*α* remain unknown, however, both p38 and AMPK, two kinases known to modify PGC-1*α* levels, are activated in response to contractile activity. Evidence suggests that p38 activation in response to exercise increases binding of MEF2 and ATF2 to the promoter of PGC-1*α* [32]. AMPK is also phosphorylated with contractile activity in skeletal muscle. Furthermore, AMPK has been shown to regulate PGC-1*α* during states of energy deprivation, such as in the case of exercise [59]. Surprisingly, exercise-induced activation of PGC-1*α* was unaltered in AMPK*α*1 and AMPK*α*2 KO mice [60]. This suggests that the induction of PGC-1*α* with exercise may be AMPK-independent, or that other kinases are able to compensate for the partial loss of AMPK. Further investigation into the initial signals mediating PGC-1*α* expression with exercise is necessary to better understand the involvement of PGC-1*α* on mitochondrial biogenesis. 

Much of the current research regarding PGC-1*α* is focused on determining whether the coactivator directly mediates exercise-induced mitochondrial adaptations. Early work suggested that PGC-1*α* may be involved, but that it does not appear to be necessary for increases in the expression of NUGEMPs in response to an endurance training regime [61]. Time course analyses revealed increased binding of NRF-1 and -2 to the cytochrome C and COXIV promoters, respectively, along with corresponding mRNA changes that precede the increased expression of PGC-1*α* [61]. These results suggest that the activation, rather than the expression of PGC-1*α* in response to exercise may be regulating the rapid initial changes in the mRNA level of NUGEMPs.

The importance of PGC-1*α* in regulating mitochondrial biogenesis in response to exercise has further been examined in PGC-1*α* transgenic animals. Research with muscle-specific PGC-1*α* overexpressing mice has demonstrated the ability of the coactivator to improve exercise performance, as revealed by greater times to exhaustion [51]. This strongly suggests the potential involvement of PGC-1*α* in exercise-induced adaptations. Findings supportive of this have also been noted in animals lacking the coactivator. PGC-1*α* KO mice have an impaired endurance capacity, as well reduced levels of key mitochondrial proteins [55]. However, skeletal muscles from PGC-1*α* KO mice display similar-fold increases in the expression of mitochondrial proteins in response to endurance training, compared to WT animals [62]. Contrary to this, muscle-specific deletion of PGC-1*α* attenuates exercise-induced mitochondrial adaptations, such as increases in cytochrome c and COXIV protein [63]. Pogozelski et al. [35] have also demonstrated that the p38*γ* MAPK-dependent increase in PGC-1*α* is required for endurance exercise-induced mitochondrial biogenesis. In addition, data from our laboratory also indicate that chronic contractile activity- (CCA-) induced levels of PGC-1*α* are necessary for the typical mitochondrial adaptations in response to contractile activity (Uguccioni, AJP). Thus, these data reveal the likelihood that multiple pathways, including PGC-1*α*-mediated gene expression, are involved in exercise-induced mitochondrial biogenesis. The identity of alternative pathways that may provoke organelle synthesis in the absence of PGC-1*α* remain to be fully discovered.

## 7. Conclusion

PGC-1*α* is a powerful coactivator of gene expression leading to mitochondrial biogenesis in muscle. Since muscle occupies a large portion of body mass, changes in mitochondrial content have a significant impact on whole body metabolism. Thus, the continued development of our understanding of the physiological and pharmacological conditions that regulate PGC-1*α* expression and function in muscle, and other tissues, is important for the optimization of whole body metabolic health.

## References

[1] Puigserver P, Spiegelman BM (2003). Peroxisome proliferator-activated receptor-*γ* coactivator 1*α* (PGC-1*α*): transcriptional coactivator and metabolic regulator. *Endocrine Reviews*.

[2] Lin J, Wu H, Tarr PT (2002). Transcriptional co-activator PGC-1*α* drives the formation of slow-twitch muscle fibres. *Nature*.

[3] Michael LF, Wu Z, Cheatham RB (2001). Restoration of insulin-sensitive glucose transporter (GLUT4) gene expression in muscle cells by the transcriptional coactivator PGC-1. *Proceedings of the National Academy of Sciences of the United States of America*.

[4] Puigserver P, Wu Z, Park CW, Graves R, Wright M, Spiegelman BM (1998). A cold-inducible coactivator of nuclear receptors linked to adaptive thermogenesis. *Cell*.

[5] Wu Z, Puigserver P, Andersson U (1999). Mechanisms controlling mitochondrial biogenesis and respiration through the thermogenic coactivator PGC-1. *Cell*.

[6] Lin J, Tarr PT, Yang R (2003). PGC-1*β* in the regulation of hepatic glucose and energy metabolism. *The Journal of Biological Chemistry*.

[7] Kressler D, Schreiber SN, Knutti D, Kralli A (2002). The PGC-1-related protein PERC is a selective coactivator of estrogen receptor *α*. *The Journal of Biological Chemistry*.

[8] Lin J, Puigserver P, Donovan J, Tarr P, Spiegelman BM (2002). Peroxisome proliferator-activated receptor *γ* coactivator 1*β* (PGC-1*β*), a novel PGC-1-related transcription coactivator associated with host cell factor. *The Journal of Biological Chemistry*.

[9] St-Pierre J, Lin J, Krauss S (2003). Bioenergetic analysis of peroxisome proliferator-activated receptor *γ* coactivators 1*α* and 1*β* (PGC-1*α* and PGC-1*β*) in muscle cells. *The Journal of Biological Chemistry*.

[10] Vercauteren K, Pasko RA, Gleyzer N, Marino VM, Scarpulla RC (2006). PGC-1-related coactivator: immediate early expression and characterization of a CREB/NRF-1 binding domain associated with cytochrome c promoter occupancy and respiratory growth. *Molecular and Cellular Biology*.

[11] Vercauteren K, Gleyzer N, Scarpulla RC (2008). PGC-1-related coactivator complexes with HCF-1 and NRF-2*β* in mediating NRF-2(GABP)-dependent respiratory gene expression. *The Journal of Biological Chemistry*.

[12] Vercauteren K, Gleyzer N, Scarpulla RC (2009). Short hairpin RNA-mediated silencing of PRC (PGC-1-related coactivator) results in a severe respiratory chain deficiency associated with the proliferation of aberrant mitochondria. *The Journal of Biological Chemistry*.

[13] Liang H, Ward WF (2006). PGC-1*α*: a key regulator of energy metabolism. *American Journal of Physiology*.

[14] Puigserver P, Adelmant G, Wu Z (1999). Activation of PPAR*γ* coactivator-1 through transcription factor docking. *Science*.

[15] Guan H-P, Ishizuka T, Chui PC, Lehrke M, Lazar MA (2005). Corepressors selectively control the transcriptional activity of PPAR*γ* in adipocytes. *Genes and Development*.

[16] Monsalve M, Wu Z, Adelmant G, Puigserver P, Fan M, Spiegelman BM (2000). Direct coupling of transcription and mRNA processing through the thermogenic coactivator PGC-1. *Molecular Cell*.

[17] Knutti D, Kaul A, Kralli A (2000). A tissue-specific coactivator of steroid receptors, identified in a functional genetic screen. *Molecular and Cellular Biology*.

[18] Esterbauer H, Oberkofler H, Krempler F, Patsch W (1999). Human peroxisome proliferator activated receptor gamma coactivator 1 (PPARGC1) gene: cDNA sequence, genomic organization, chromosomal localization, and tissue expression. *Genomics*.

[19] Irrcher I, Adhihetty PJ, Sheehan T, Joseph A-M, Hood DA (2003). PPAR*γ* coactivator-1*α* expression during thyroid hormone- and contractile activity-induced mitochondrial adaptations. *American Journal of Physiology*.

[20] Adhihetty PJ, Uguccioni G, Leick L, Hidalgo J, Pilegaard H, Hood DA (2009). The role of PGC-1*α* on mitochondrial function and apoptotic susceptibility in muscle. *American Journal of Physiology*.

[21] Vega RB, Huss JM, Kelly DP (2000). The coactivator PGC-1 cooperates with peroxisome proliferator-activated receptor *α* in transcriptional control of nuclear genes encoding mitochondrial fatty acid oxidation enzymes. *Molecular and Cellular Biology*.

[22] Falkenberg M, Larsson N-G, Gustafsson CM (2007). DNA replication and transcription in mammalian mitochondria. *Annual Review of Biochemistry*.

[23] Wallace DC (2005). A mitochondrial paradigm of metabolic and degenerative diseases, aging, and cancer: a dawn for evolutionary medicine. *Annual Review of Genetics*.

[24] Hood DA, Irrcher I, Ljubicic V, Joseph A-M (2006). Coordination of metabolic plasticity in skeletal muscle. *Journal of Experimental Biology*.

[25] Gleyzer N, Vercauteren K, Scarpulla RC (2005). Control of mitochondrial transcription specificity factors (TFB1M and TFB2M) by nuclear respiratory factors (NRF-1 and NRF-2) and PGC-1 family coactivators. *Molecular and Cellular Biology*.

[26] Scarpulla RC (2006). Nuclear control of respiratory gene expression in mammalian cells. *Journal of Cellular Biochemistry*.

[27] Schreiber SN, Emter R, Hock MB (2004). The estrogen-related receptor *α* (ERR*α*) functions in PPAR*γ* coactivator 1*α* (PGC-1*α*)-induced mitochondrial biogenesis. *Proceedings of the National Academy of Sciences of the United States of America*.

[28] Narkar VA, Downes M, Yu RT (2008). AMPK and PPAR*δ* agonists are exercise mimetics. *Cell*.

[29] Wang Y-X, Zhang C-L, Yu RT (2004). Regulation of muscle fiber type and running endurance by PPAR*δ*. *PLoS Biology*.

[30] Ljubicic V, Joseph A-M, Saleem A (2010). Transcriptional and post-transcriptional regulation of mitochondrial biogenesis in skeletal muscle: effects of exercise and aging. *Biochimica et Biophysica Acta*.

[31] Pilegaard H, Saltin B, Neufer DP (2003). Exercise induces transient transcriptional activation of the PGC-1*α* gene in human skeletal muscle. *Journal of Physiology*.

[32] Akimoto T, Pohnert SC, Li P (2005). Exercise stimulates Pgc-1*α* transcription in skeletal muscle through activation of the p38 MAPK pathway. *The Journal of Biological Chemistry*.

[33] Yan Z, Li P, Akimoto T (2007). Transcriptional control of the Pgc-1*α* gene in skeletal muscle in vivo. *Exercise and Sport Sciences Reviews*.

[34] Ojuka EO, Jones TE, Han D-H, Chen M, Holloszy JO (2003). Raising Ca^2+^ in L6 myotubes mimics effects of exercise on mitochondrial biogenesis in muscle. *FASEB Journal*.

[35] Pogozelski AR, Geng T, Li P (2009). p38*γ* mitogen-activated protein kinase is a key regulator in skeletal muscle metabolic adaptation in mice. *PLoS ONE*.

[36] Irrcher I, Ljubicic V, Kirwan AF, Hood DA (2008). AMP-activated protein kinase-regulated activation of the PGC-1*α* promoter in skeletal muscle cells. *PLoS ONE*.

[37] Irrcher I, Ljubicic V, Hood DA (2009). Interactions between ROS and AMP kinase activity in the regulation of PGC-1*α* transcription in skeletal muscle cells. *American Journal of Physiology*.

[38] Amat R, Planavila A, Chen SL, Iglesias R, Giralt M, Villarroya F (2009). SIRT1 controls the transcription of the peroxisome proliferator-activated receptor-*γ* co-activator-1*α*(PGC-1*α*) gene in skeletal muscle through the PGC-1*α* autoregulatory loop and interaction with MyoD. *The Journal of Biological Chemistry*.

[39] Handschin C, Rhee J, Lin J, Tarr PT, Spiegelman BM (2003). An autoregulatory loop controls peroxisome proliferator-activated receptor *γ* coactivator 1*α* expression in muscle. *Proceedings of the National Academy of Sciences of the United States of America*.

[40] Puigserver P, Rhee J, Lin J (2001). Cytokine stimulation of energy expenditure through p38 MAP kinase activation of PPAR*γ* coactivator-1. *Molecular Cell*.

[41] Li X, Monks B, Ge Q, Birnbaum MJ (2007). Akt/PKB regulates hepatic metabolism by directly inhibiting PGC-1*α* transcription coactivator. *Nature*.

[42] Fan M, Rhee J, St-Pierre J (2004). Suppression of mitochondrial respiration through recruitment of p160 myb binding protein to PGC-1*α*: modulation by p38 MAPK. *Genes and Development*.

[43] Knutti D, Kressler D, Kralli A (2001). Regulation of the transcriptional coactivator PGC-1 via MAPK-sensitive interaction with a repressor. *Proceedings of the National Academy of Sciences of the United States of America*.

[44] Jager S, Handschin C, St-Pierre J, Spiegelman BM (2007). AMP-activated protein kinase (AMPK) action in skeletal muscle via direct phosphorylation of PGC-1*α*. *Proceedings of the National Academy of Sciences of the United States of America*.

[45] Lerin C, Rodgers JT, Kalume DE, Kim S-H, Pandey A, Puigserver P (2006). GCN5 acetyltransferase complex controls glucose metabolism through transcriptional repression of PGC-1*α*. *Cell Metabolism*.

[46] Nemoto S, Fergusson MM, Finkel T (2005). SIRT1 functionally interacts with the metabolic regulator and transcriptional coactivator PGC-1*α*. *The Journal of Biological Chemistry*.

[47] Rodgers JT, Lerin C, Haas W, Gygi SP, Spiegelman BM, Puigserver P (2005). Nutrient control of glucose homeostasis through a complex of PGC-1*α* and SIRT1. *Nature*.

[48] Lagouge M, Argmann C, Gerhart-Hines Z (2006). Resveratrol improves mitochondrial function and protects against metabolic disease by activating SIRT1 and PGC-1*α*. *Cell*.

[49] Heun P (2007). SUMOrganization of the nucleus. *Current Opinion in Cell Biology*.

[50] Rytinki MM, Palvimo JJ (2009). SUMOylation attenuates the function of PGC-1*α*. *The Journal of Biological Chemistry*.

[51] Calvo JA, Daniels TG, Wang X (2008). Muscle-specific expression of PPAR*γ* coactivator-1*α* improves exercise performance and increases peak oxygen uptake. *Journal of Applied Physiology*.

[52] Wende AR, Schaeffer PJ, Parker GJ (2007). A role for the transcriptional coactivator PGC-1*α* in muscle refueling. *The Journal of Biological Chemistry*.

[53] Leone TC, Lehman JJ, Finck BN (2005). PGC-1alpha deficiency causes multi-system energy metabolic derangements: muscle dysfunction, abnormal weight control and hepatic steatosis. *PLoS Biology*.

[54] Lin J, Wu P-H, Tarr PT (2004). Defects in adaptive energy metabolism with CNS-linked hyperactivity in PGC-1*α* null mice. *Cell*.

[55] Handschin C, Chin S, Li P (2007). Skeletal muscle fiber-type switching, exercise intolerance, and myopathy in PGC-1*α* muscle-specific knock-out animals. *The Journal of Biological Chemistry*.

[56] Goto M, Terada S, Kato M (2000). cDNA cloning and mRNA analysis of PGC-1 in epitrochlearis muscle in swimming-exercised rats. *Biochemical and Biophysical Research Communications*.

[57] Connor MK, Irrcher I, Hood DA (2001). Contractile activity-induced transcriptional activation of cytochrome C involves Sp1 and is proportional to mitochondrial ATP synthesis in C2C12 muscle cells. *The Journal of Biological Chemistry*.

[58] Gordon JW, Rungi AA, Inagaki H, Hood DA (2001). Selected contribution: effects of contractile activity on mitochondrial transcription factor A expression in skeletal muscle. *Journal of Applied Physiology*.

[59] Zong H, Ren JM, Young LH (2002). AMP kinase is required for mitochondrial biogenesis in skeletal muscle in response to chronic energy deprivation. *Proceedings of the National Academy of Sciences of the United States of America*.

[60] Jørgensen SB, Wojtaszewski JFP, Viollet B (2005). Effects of *α*-AMPK knockout on exercise-induced gene activation in mouse skeletal muscle. *FASEB Journal*.

[61] Wright DC, Han D-H, Garcia-Roves PM, Geiger PC, Jones TE, Holloszy JO (2007). Exercise-induced mitochondrial biogenesis begins before the increase in muscle PGC-1*α* expression. *The Journal of Biological Chemistry*.

[62] Leick L, Wojtaszewski JFP, Johansen ST (2008). PGC-1*α* is not mandatory for exercise- and training-induced adaptive gene responses in mouse skeletal muscle. *American Journal of Physiology*.

[63] Geng T, Li P, Okutsu M (2010). PGC-1*α* plays a functional role in exercise-induced mitochondrial biogenesis and angiogenesis but not fiber-type transformation in mouse skeletal muscle. *American Journal of Physiology*.

